# Regular Supplementation With Resveratrol Improves Bone Mineral Density in Postmenopausal Women: A Randomized, Placebo‐Controlled Trial

**DOI:** 10.1002/jbmr.4115

**Published:** 2020-07-14

**Authors:** Rachel HX Wong, Jay Jay Thaung Zaw, Cory J Xian, Peter RC Howe

**Affiliations:** ^1^ School of Biomedical Sciences and Pharmacy University of Newcastle Callaghan Australia; ^2^ Institute for Resilient Regions University of Southern Queensland Springfield Central Australia; ^3^ UniSA Clinical & Health Sciences and Cancer Research Institute University of South Australia Adelaide Australia; ^4^ UniSA Allied Health & Human Performance University of South Australia Adelaide Australia

**Keywords:** AGING, CLINICAL TRIALS, DXA, MENOPAUSE, NUTRITION

## Abstract

Resveratrol, a naturally occurring polyphenol in red grapes and berries, can act as a phytoestrogen. It has been shown to improve both systemic and cerebral circulatory functions, possibly through activation of endothelial estrogen receptors. in vitro and in vivo studies in rodent models also indicate a bone‐protective role for resveratrol, particularly in ovariectomized rat models that mimic postmenopausal osteoporosis caused by estrogen deficiency. Hypothesizing a circulatory benefit of resveratrol in bone tissue, we investigated whether resveratrol supplementation could improve bone health in postmenopausal women. The Resveratrol for Healthy Aging in Women (RESHAW) trial was a 24‐month randomized, double‐blind, placebo‐controlled, two‐period crossover intervention conducted to evaluate the effects of resveratrol (75 mg twice daily) on cognition, cerebrovascular function, bone health, cardiometabolic markers, and well‐being in postmenopausal women. After 12 months of supplementation with resveratrol versus placebo, there were positive effects on bone density in the lumbar spine (+0.016 ± 0.003 g/cm^2^) and neck of femur (+0.005 ± 0.002 g/cm^2^), which were accompanied by a 7.24% reduction in C‐terminal telopeptide type‐1 collagen levels, a bone resorption marker, compared with placebo. The increase in bone mineral density in the femoral neck resulted in an improvement in *T*‐score (+0.070 ± 0.018) and a reduction in the 10‐year probability of major and hip fracture risk. The magnitude of improvement was higher in women with poor bone health biomarker status. Importantly, the improvement in femoral neck *T*‐score with resveratrol correlated with improvement in perfusion. Our subanalysis also revealed that the bone‐protective benefit of resveratrol was greater in participants who supplemented with vitamin D plus calcium. Regular supplementation with 75 mg of resveratrol twice daily has the potential to slow bone loss in the lumbar spine and femoral neck, common fracture sites in postmenopausal women without overt osteoporosis. © 2020 The Authors. *Journal of Bone and Mineral Research* published by American Society for Bone and Mineral Research.

## Introduction

Osteoporosis is a silent disease characterized by progressive deterioration of bone tissue, gradually compromising bone strength. Unsuspecting individuals are often confronted with a detrimental hip fracture resulting from a fall or suffer from long‐standing back pain caused by crushed vertebrae. The two most common forms of primary osteoporosis in the elderly are postmenopausal osteoporosis (type 1) and senile osteoporosis (type 2). The powerful ability of estrogen to suppress osteoclast activity in the resorption phase and enhance its apoptosis is lost with estrogen depletion at menopause. Trabecular bone is especially affected in postmenopausal osteoporosis where women lose an impressive 12% of bone mass within 5 to 7 years, an equivalent of one *T*‐score measured by dual‐energy X‐ray absorptiometry (DXA).^(^ ^1^, ^2^ ^)^ The greater loss of trabecular than cortical bone predisposes individuals to low‐trauma vertebral fractures that remain undetected.^(^ ^3^ ^)^ Senile osteoporosis is caused by age‐related bone loss associated with decreased proliferation of osteogenic stem cells, which slows bone formation.^(^ ^4^, ^5^ ^)^ Elderly postmenopausal women are likely to have both etiologies.

Increasingly, evidence suggests that intraosseous blood vessels serve as a source for mesenchymal stem cells to induce their differentiation into osteoblasts for new bone formation.^(^ ^6^ ^)^ Aging decreases the quality of the blood supply to body tissues, and it can further compromise bone perfusion in the presence of chronic diseases. Hypoxia upregulates osteoclast activities and inhibits osteoblast as shown in in vitro studies,^(^ ^7^, ^8^ ^)^ which may account for the consistent epidemiological link between osteoporosis and ischemic vascular diseases.^(^ ^9^ ^)^ Addressing microvascular dysfunction may represent an additional pathway to counteract future osteoporosis.

Phytoestrogens such as soy isoflavones and resveratrol have structural similarity to estrogen and can bind to estrogen receptors to exert a multitude of benefits for which estrogen is responsible, and they have attracted interest as potential bone health therapies in estrogen‐deficient postmenopausal women. A 24‐month randomized controlled trial in a large cohort of osteopenic postmenopausal women provided strong evidence of a clinical indication for soy isoflavones on bone health. There was a progressive increase in bone mineral density (BMD) of the femoral neck rising from 3.5% at 12 months up to 9.3% by the end of the trial with a daily supplement of 56 mg genistein, 500 mg calcium carbonate, and 400 IU vitamin D compared with placebo.^(^ ^10^ ^)^ The BMD changes were attributed to the increase in osteoprotegerin, a soluble decoy receptor for receptor activator of NF‐κB ligand (RANKL) that inhibits differentiation and function of osteoclasts.^(^ ^11^ ^)^ Soy isoflavones have been shown to improve endothelial function; however, no studies have linked the bone benefits with improved vascular function.^(^ ^12^ ^)^

Resveratrol is a stilbene found on skins of red grapes and berries and has been shown in animal models to promote osteoblast‐mediated bone formation and inhibit osteoclast‐stimulated bone resorption via similar mechanisms to genistein.^(^ ^13^ ^)^ To date, only three clinical studies have evaluated the effects of resveratrol on BMD, but their results were lackluster.^(^ ^14^, ^15^, ^16^ ^)^ None of the studies supplemented participants for more than 6 months or were undertaken in postmenopausal women to see whether estrogenic effects of resveratrol can counteract postmenopausal osteoporosis. The cardiovascular benefits of resveratrol, on the other hand, are well established.^(^ ^17^ ^)^ In an acute dose–response study in adults with type 2 diabetes, we found that 75 mg resveratrol was equally or more efficacious than 150 mg and 300 mg doses for enhancing cerebral vasodilatation and improving sustained attention.^(^ ^18^, ^19^ ^)^ In a follow‐up study, we have reported improvements of cerebrovascular function in postmenopausal women after 14 weeks of resveratrol supplementation.^(^ ^20^ ^)^ The benefits of resveratrol for cerebrovascular function might also be partially mediated by the activation of estrogen receptors on endothelial cells to facilitate vasodilatation.^(^ ^21^ ^)^ It is plausible that the ability of resveratrol to enhance microvascular perfusion may also benefit bone health, especially in postmenopausal women. In the present study, we investigated whether resveratrol has beneficial effects on BMD in postmenopausal women and, if so, whether there is any potential interaction with vitamin D and/or calcium supplements as older adults often consume them. In addition, we aimed to elucidate whether any treatment changes in our measured bone parameters correlate with resveratrol‐induced improvements in vascular function.

## Materials and Methods

### Study design

The Resveratrol for Healthy Aging in Women (RESHAW) trial is a 24‐month randomized, double‐blind, placebo‐controlled, two‐period crossover intervention conducted to evaluate the effects of resveratrol supplementation (75 mg twice daily) on cognitive performance, cerebrovascular function, bone health, cardiometabolic markers, and well‐being in postmenopausal women. The outcomes for cognitive performance, cerebrovascular function, and cardiometabolic markers are published elsewhere.^(^ ^22^ ^)^ This article now reports outcomes for bone health and biomarkers of bone metabolism.

RESHAW was conducted at the Clinical Nutrition Research Centre of the University of Newcastle, Australia, in accordance with the Declaration of Helsinki and the Principles of Good Clinical Practice as outlined by the International Conference on Harmonization. Written informed consent was obtained before any assessment. The protocol was approved by the University of Newcastle's Human Research Ethics Committee (H‐2016‐0091) and registered with the Australia and New Zealand Clinical Trials Registry (ACTRN12616000679482p).

### Participants

Between November 2016 and June 2017, community‐dwelling women from the Hunter region of New South Wales in Australia were recruited through approved newspaper and radio campaigns and from a database of previous participation in our Centre's trials and the Hunter Medical Research Volunteers Registry. To be included in the study, participants were aged 45 to 85 years, more than 12 months of cessation of menses, and not taking hormone replacement therapy. We excluded participants who took insulin or warfarin or had a history of breast or cervical cancer, major heart, kidney or liver disease, a neurological disorder, clinical depression, or suspected dementia.

### Intervention and randomization

Resveratrol (Veri‐te resveratrol) capsules (containing 75 mg of >98% of *trans*‐resveratrol) and placebo capsules (comprising several inert excipients) of identical shape and color were supplied by Evolva SA (Reinach, Switzerland). Capsules were dispensed in containers identifiable only by code numbers. An independent investigator who was not involved in recruitment or data collection held the code and allocated volunteers to resveratrol or placebo capsules using Altman's randomization by minimization procedure,^(^ ^23^ ^)^ balancing the treatment groups based on age, menopausal years, and clinic blood pressure at the screening visit. All other investigators and participants were blinded to the supplement allocation throughout data collection and analysis.

Participants were instructed to take two capsules of their allocated treatment each day (one in the morning and one in the evening) for 12 months, after which they crossed over to the alternate treatment for a further 12 months. Participants were also encouraged to maintain their habitual diet, including other dietary supplements, and physical activity levels throughout the 2‐year study.

### Bone density and bone metabolism assessments

We assessed the participants' bone density and blood markers of bone metabolism at baseline and the end of each treatment phase. Participants attended the clinic in the mornings after an overnight fasting of more than 8 hours and voided their bladders before changing into a disposable paper gown. Using a Lunar Prodigy densitometer, areal BMD was measured by DXA in the lumbar spine (L_1_ to L_4_), left and right hip (total hip and neck of femur) and the whole body in an anterior–posterior plane (GE Healthcare Lunar, Madison, WI, USA). Baseline and follow‐up scans were obtained with the same instrument. On the dates of data acquisition, at least 10 phantom scans were performed in accordance with the manufacturer's instructions to ensure that the densitometer stability and BMD measurement precision remained within 0.4% from the reference value. Between November 2017 and June 2019, the instrument precision was 0.267%. Licensed technicians (ie, authors RHXW and JJTZ) obtained all scans on the participants. The precision of repositioning at our site expressed as the root mean square of the percent coefficient of variation (RMS‐% CV) was 0.52% for the spine, 0.39% for femur neck, 0.51% for total hip, and 0.26% for the whole body. The integrated software (GE Healthcare Encore software v16) was used to generate the BMD, *T*‐scores, and *Z*‐scores for the lumbar spine, hip, and whole body. Region‐of‐interest in the spine, hips, or whole body scans were manually adjusted and excluded if necessary on scans showing metal artifacts or breast implants or severe scoliosis and/or severe degenerative disc disease. In participants with a hip replacement, the contralateral hip was used.

The BMD values of the dual neck of femur obtained with the DXA and the participants' relevant medical history were entered into the Fracture Risk Assessment Tool (FRAX) calculator for Australia region to obtain participants' *T*‐scores at the hip and the 10‐year probability of sustaining a major osteoporotic fracture and hip fracture (https://www.sheffield.ac.uk/FRAX/tool.aspx?country=31).

Venous blood samples were obtained immediately after their DXA scans. Blood samples were centrifuged at 4000*g* for 10 minutes at 4°C and the plasma was divided into aliquots and stored at −80°C within 2 hours of collection. Pathology New South Wales, a commercial pathology laboratory in the Hunter region of New South Wales, assessed the samples for plasma levels of osteocalcin and C‐terminal telopeptide type‐1 collagen (CTX), markers of bone formation and bone resorption, respectively.

### Cerebral vasodilator responsiveness to a hypercapnic challenge

A detailed description of the methodology for cerebrovascular responsiveness (CVR) to hypercapnia assessment can be found in our previous publications.^(^ ^20^, ^22^ ^)^ Briefly, participants fasted at least 2 hours before breathing in a carbogen gas mix (95% O_2_/5% CO_2_) for 3 minutes. Transcranial Doppler ultrasound was used to detect changes in mean blood flow velocity of the middle cerebral arteries. The percent change from mean blood flow velocity at rest to the peak velocity denotes the CVR, which is an indirect measure of maximum global cerebral perfusion capacity in response to the metabolic demand. Higher values of CVR reflect superior cerebrovasodilator capacity. This marker is used as a surrogate measure of overall vascular function.

### Sample size and statistical analysis

The sample size for the RESHAW trial was calculated based on the primary outcome, which was the mean within‐individual difference in overall cognitive performance between resveratrol and placebo treatment phases. For 90% power to detect a statistically significant (*p* < 0.05) medium effect size (Cohen's *d* = 0.5) improvement in the primary outcome, 87 completers were required in the crossover comparison. To allow for 45% attrition due to a long‐term study, we aimed to recruit 170 women. This article will also report a parallel comparison analysis performed at the end of the first 12‐month phase of the crossover to compare mean treatment changes (post‐pre supplementation) between the placebo and resveratrol arms. For 80% power to detect a statistically significant (*p* < 0.05) medium effect size in the parallel comparison, 64 people per arm were required.

For the parallel analysis, an independent *t* test was used to compare the change from baseline between the placebo group and the resveratrol group. Within‐individual treatment effects at the end of each supplementation period were compared using repeated measures ANOVA (analysis of variance). The Benjamini‐Hochberg procedure^(^ ^24^ ^)^ was used to correct *p* values for all variables in this analysis as they were secondary outcomes in the RESHAW trial to minimize type 1 errors; the false discovery rate was set at 0.15 unless otherwise stated.

We undertook an exploratory subgroup analysis to identify potential interactions with the resveratrol treatment according to participants' baseline bone density *T*‐score bone health classification in the hip and regular use of vitamin D and/or calcium supplements. Pearson's correlation was used to test for a relationship between treatment change in CVR and changes in bone parameters (ie, BMD and *T*‐scores). All tests in the exploratory analysis were two‐tailed and *p* < 0.05 was considered significant.

All analyses were performed with SPSS version 25.0 (SPSS by IBM Inc., Chicago, IL, USA). Data are presented as mean ± SEM (standard error of the mean).

## Results

### Participant characteristics

**Fig 1:**
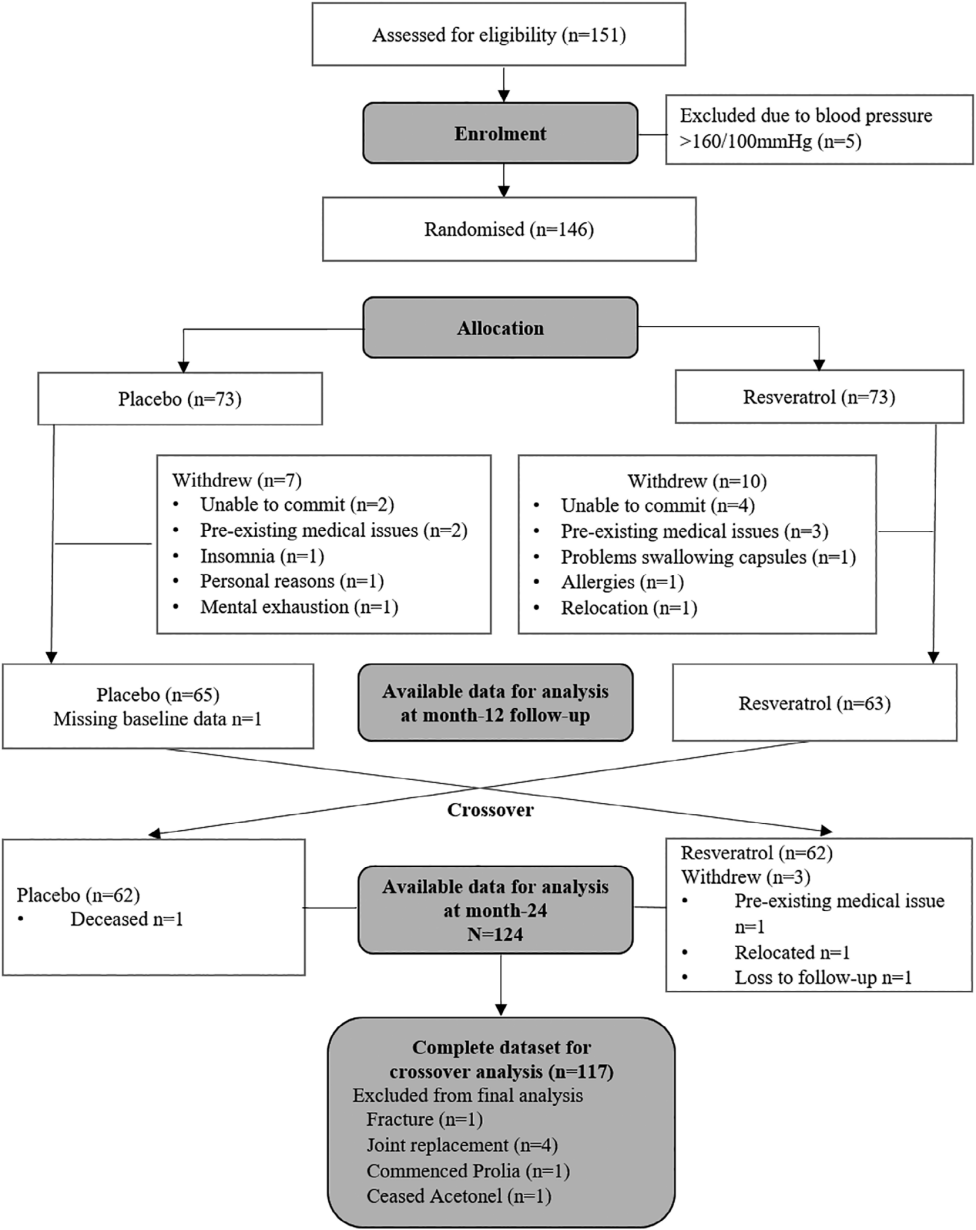
Consort diagram. Flow of participants from initial contact to final follow‐up and available data for parallel analysis and crossover comparison.

The flow of participants is depicted in Fig. 1. At the end of the first 12‐month intervention phase, 66 women in the placebo group and 63 in the resveratrol group returned to the clinic for reassessments of outcome measures, and 125 women completed the 2‐year crossover trial by June 2019. Details of the adverse events have been reported.^(^ ^22^ ^)^ Briefly, of the 12 adverse events reported, 4 occurred in the resveratrol group but were not necessarily attributed to supplementation (viz., itching, menses, prolapsed bladder, and prescheduled eye operation). Based on capsule counts, overall compliance was 95% and 94% for placebo and resveratrol groups, respectively, over the first 12 months of the intervention. For the crossover analysis, the average compliance was 96% for both groups.

In the crossover analysis, we excluded data from 7 participants because they had sustained a fracture (*n* = 1), had joint replacement surgery (*n* = 4), commenced denosumab (Prolia) therapy (*n* = 1), or ceased risedronic acid (Acetonel) therapy (*n* = 1) after study enrollment. We also excluded lumbar spine data from 2 participants because of severe spinal stenosis or awaiting surgery upon completion of the 2‐year study.

The women in the study were marginally overweight but normotensive. Based on their femoral neck *T*‐scores at enrollment, our cohort was mildly osteopenic (defined as *T*‐score between −1.0 and − 2.5). Specifically, 50 women had normal bone density, 72 women had osteopenia, and 6 women had osteoporosis in the hips (*T*‐score < −2.5). One participant had a double‐hip joint replacement; her lumbar spine *T*‐score was used instead for this classification. Baseline characteristics did not differ significantly between those allocated to placebo and resveratrol at enrollment (Table 1).

**Table 1:** Demographic and Bone Health Status at Baseline of Participants Who Completed the First Intervention Phase of the Study

Characteristics	Placebo (*n* = 65)	Resveratrol (*n* = 63)	*p* Value
Age (years)	65.8 ± 1.3	64.3 ± 1.3	0.772
Menopausal years	15.6 ± 1.1	15.5 ± 1.2	0.947
Body mass index (kg/m^2^)	25.7 ± 0.5	25.6 ± 0.5	0.867
Systolic BP (mmHg)	125 ± 2	124 ± 2	0.743
Diastolic BP (mmHg)	69 ± 1	68 ± 1	0.529
Pulse rate (bpm)	61 ± 1	62 ± 1	0.740
Lumbar spine			
BMD (g/cm^2^)	1.085 ± 0.019	1.088 ± 0.021	0.916
*T*‐score	−1.08 ± 0.11	−1.13 ± 0.11	0.749
*Z*‐score	0.59 ± 0.17	0.62 ± 0.12	0.801
Neck of femur			
BMD (g/cm^2^)	0.886 ± 0.015	0.881 ± 0.013	0.836
*T*‐score	−1.09 ± 0.11	−1.13 ± 0.10	0.764
*Z*‐score	0.51 ± 0.12	0.53 ± 0.10	0.919
Total hip			
BMD (g/cm^2^)	0.916 ± 0.016	0.919 ± 0.011	0.785
*T*‐score	−0.69 ± 0.12	−0.75 ± 0.12	0.714
*Z*‐score	0.44 ± 0.13	0.43 ± 0.09	0.923
10‐year major fracture risk (%)	4.7 ± 0.5	5.5 ± 0.5	0.263
10‐year hip fracture risk (%)	1.1 ± 0.3	1.6 ± 0.3	0.272
Total body BMD (g/cm^2^)	1.122 ± 0.014	1.129 ± 0.015	0.768
Osteocalcin (μg/L)	20.5 ± 0.7	20.8 ± 0.8	0.764
CTX (ng/L)	423.3 ± 24.2	467.4 ± 22.4	0.184
Never took supplements (*n*)	48	49	—
Vitamin D only (*n*)	7	9	—
Calcium only (*n*)	2	0	—
Vitamin D and calcium (*n*)	8	5	—

BP = blood pressure; bpm = beats per minute; BMD = bone mineral density; CTX = C‐terminal telopeptide type‐1 collagen.

Baseline profiles of the bone health parameters of participants who did not take any vitamin D or calcium supplements and those who took vitamin D only, calcium only, or both are presented in Supplemental Table S1. There were no significant differences in age between participants who took vitamin D and/or calcium supplements and those who took neither supplement. At baseline, participants who were regularly consuming vitamin D only had greater BMD in the lumbar spine compared with those who took neither supplement. Participants who consumed both vitamin D and calcium had significantly lower BMD and *T*‐score in the neck of femur (−8.5%) compared with those who took neither supplement (Supplemental Table S1). Four participants were prescribed Prolia and remained on treatment throughout the study. In addition to their bone‐active medication, one participant regularly consumed vitamin D only, one participant consumed calcium only, another participant consumed both supplements, and one participant took neither.

### Cohort data (parallel analysis)

**Fig 2:**
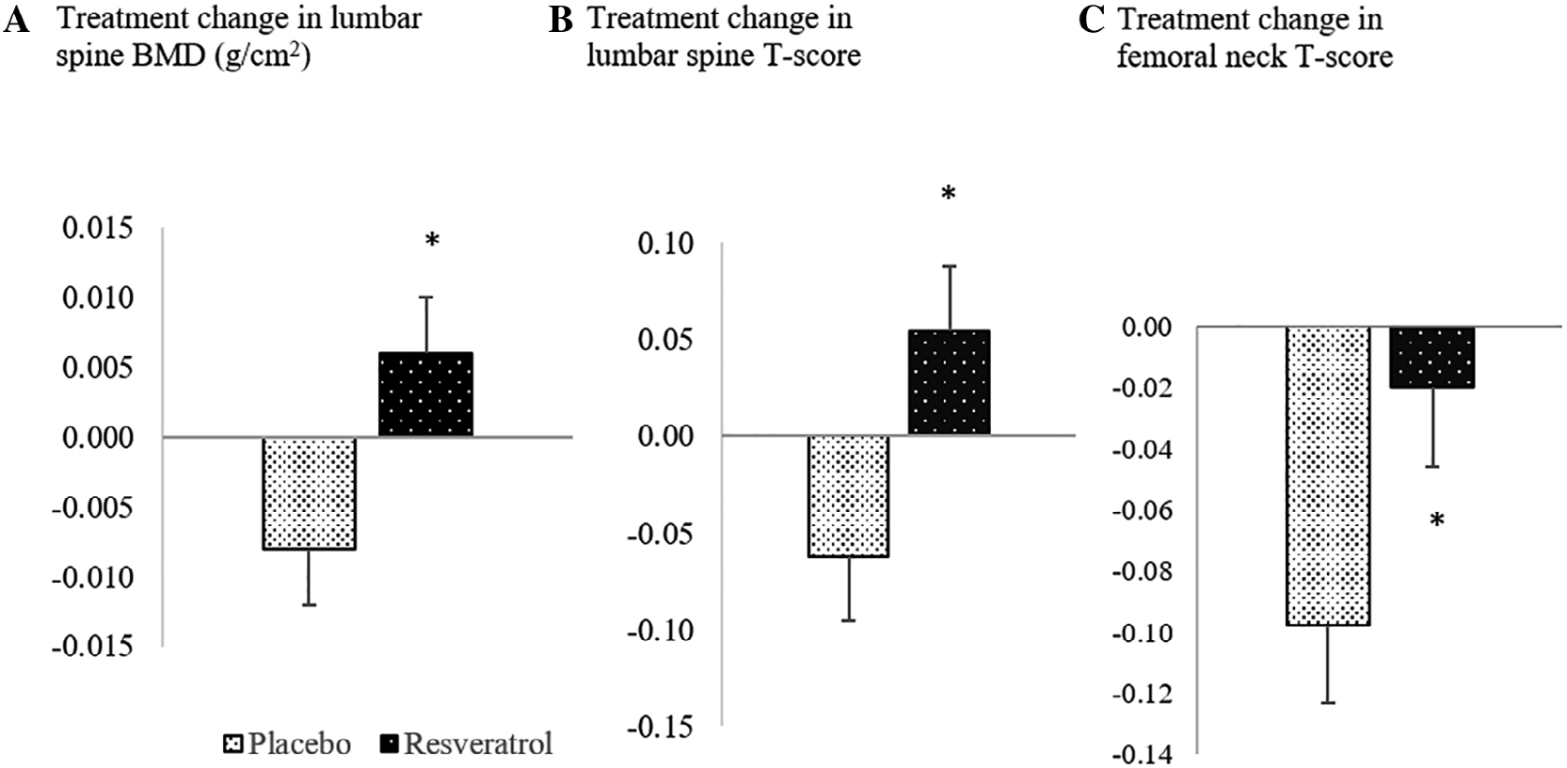
Treatment changes from baseline values of (*A*) lumbar spine bone mineral density (BMD), (*B*) lumbar spine *T*‐score, and (*C*) femoral neck *T*‐score. * indicates q* < 0.15.

Table 2 presents bone health status after 12 months of placebo or resveratrol supplementation. There was no difference in whole body BMD after resveratrol supplementation compared with placebo. However, relative to placebo, BMD increased in the lumbar spine by a modest 1.3% (q* = 0.049), thus improving the mean *T*‐score by 1.5% (q* = 0.079) (Fig. 2). Likewise, BMD and corresponding *T*‐scores in the neck of femur and total hip were also increased by resveratrol relative to placebo, which in turn significantly attenuated the 10‐year probability of sustaining a major osteoporotic fracture or hip fracture. There was a trend toward a reduction of CTX after 12 months of resveratrol supplementation (∆placebo: −7.35 ± 13.72 ng/L versus ∆resveratrol: −39.04 ± 13.46 ng/L). Plasma osteocalcin levels were unaffected by supplementation.

**Table 2:** Outcomes of Bone Health After 12 Months of Placebo or Resveratrol Supplementation in the Parallel Comparison

	Placebo	Resveratrol	∆resveratrol‐∆placebo [95% CI]	*p* Value (absolute)	q* (FDR)
Lumbar spine	*n* = 65	*n* = 61			q < 0.15
BMD (g/cm^2^)	1.073 ± 0.018	1.090 ± 0.02	0.014 ± 0.005 [0.004, 0.024]	0.007	**0.049**
*T*‐score	−0.841 ± 0.159	−0.702 ± 0.165	0.116 ± 0.048 [0.042, 0.227]	0.017	**0.079**
*Z*‐score	0.592 ± 0.164	0.723 ± 0.170	0.074 ± 0.050 [0.025, 0.203]	0.140	0.178
Femur and total hip	*n* = 65	*n* = 63			
Neck BMD (g/cm^2^)	0.877 ± 0.015	0.878 ± 0.014	0.009 ± 0.005 [−0.001, 0.019]	0.065	**0.137**
Neck *T*‐score	−1.161 ± 0.107	−1.148 ± 0.098	0.075 ± 0.038 [−0.003, 0.142]	0.047	**0.121**
Neck *Z*‐score	0.506 ± 0.117	0.917 ± 0.016	0.079 ± 0.043 [−0.008, 0.158]	0.068	**0.136**
Total hip (g/cm^2^)	0.917 ± 0.016	0.913 ± 0.016	0.006 ± 0.004 [−0.006, 0.012]	0.200	**0.137**
Total hip *T*‐score	−0.721 ± 0.131	−0.755 ± 0.128	0.028 ± 0.037 [−0.051, 0.093]	0.441	0.475
Total hip *Z*‐score	0.445 ± 0.136	0.460 ± 0.132	0.037 ± 0.038 [−0.043, 0.107]	0.322	0.376
Major fracture risk (%)	5.62 ± 0.61	5.17 ± 0.45	−0.376 ± 0.192 [−0.755, 0.004]	0.052	**0.121**
Hip fracture risk (%)	1.56 ± 0.39	1.51 ± 0.28	−0.432 ± 0.208 [−0.844, −0.020]	0.040	**0.121**
Whole body	*n* = 65	*n* = 63			
Total BMD (g/cm^2^)	1.117 ± 0.014	1.124 ± 0.015	0.007 ± 0.003 [−0.006, 0.007]	0.871	0.871
Osteocalcin (μg/L)	19.5 ± 0.9	19.3 ± 0.8	−1.091 ± 0.951 [−2.972, 0.790]	0.253	0.656
CTX (ng/L)	404.5 ± 23.5	427.5 ± 19.2	−31.69 ± 19.22 [−69.81, 6.43]	0.098	**0.137**

CI = confidence interval; FDR = false discovery rate; BMD = bone mineral density; CTX = C‐terminal telopeptide type‐1 collagen.

Q* (FDR) is the level of significance after applying false discovery rate of 0.15 to the cohort's data.

### Cohort data (crossover analysis)

As shown in Table 3, our crossover analysis confirms the results of the 12‐month parallel analysis. Compared with the placebo treatment, the improvement in BMD at the lumbar spine and neck of femur and femoral neck *T*‐score remained significantly higher after resveratrol treatment. In addition, CTX was now significantly lower than placebo. However, major osteoporotic fracture and hip fracture risks were not significantly different between the treatments. Furthermore, the improvement in total hip BMD was no longer significant because it exceeded the precision error threshold. Whole body BMD and osteocalcin levels remained unaffected with resveratrol supplementation.

**Table 3:** Crossover Comparisons of Bone Mineral Density of Lumbar Spine, Femur, Total Hip, and Whole Body After Placebo and Resveratrol Supplementation

	Baseline	Placebo	Resveratrol	Resveratrol‐placebo [95% CI]	*p* Value (absolute)	q* (FDR)
Lumbar spine (*n* = 115)						q < 0.15
BMD (g/cm^2^)	1.089 ± 0.013	1.069 ± 0.015	1.085 ± 0.014	0.016 ± 0.003 [0.009, 0.022]	<0.001	**<0.001**
*T*‐score	−0.75 ± 0.11	−1.02 ± 0.11	−0.87 ± 0.11	0.151 ± 0.028 [0.096, 0.207]	<0.001	**<0.001**
*Z*‐score	0.62 ± 0.11	0.44 ± 0.12	0.58 ± 0.11	0.144 ± 0.026 [0.092, 0.196]	<0.001	**<0.001**
Femur and total hip (*n* = 117)						
Neck BMD (g/cm^2^)	0.883 ± 0.010	0.870 ± 0.011	0.875 ± 0.011	0.005 ± 0.002 [0.000, 0.010]	0.035	**0.058**
Neck *T*‐score	−1.11 ± 0.07	−1.20 ± 0.075	−1.17 ± 0.075	0.035 ± 0.018 [6.968 x 10^−5^, 0.069]	0.050	**0.068**
Neck *Z*‐score	0.52 ± 0.08	0.49 ± 0.08	0.53 ± 0.09	0.047 ± 0.019 [0.009, 0.085]	0.016	**0.034**
Total hip (g/cm^2^)	0.917 ± 0.011	0.908 ± 0.012	0.911 ± 0.012	0.004 ± 0.002 [0.000, 0.007]	0.029	**0.054**
Total hip *T*‐score	−0.72 ± 0.09	−0.80 ± 0.10	−0.77 ± 0.10	0.027 ± 0.014 [1.907 x 10^−6^, 0.054]	0.050	**0.068**
Total hip *Z*‐score	0.43 ± 0.09	0.41 ± 0.10	0.45 ± 0.10	0.036 ± 0.013 [0.009, 0.062]	0.009	**0.023**
Major fracture risk (%)	5.1 ± 0.4	5.8 ± 0.4	5.7 ± 0.4	−0.047 ± 0.172 [0.388, 0.295]	0.787	0.787
Hip fracture risk (%)	1.3 ± 0.2	1.7 ± 0.2	1.6 ± 0.2	−0.115 ± 0.181 [−0.473, 0.243]	0.525	0.656
Whole body BMD (*n* = 117)						
Whole body BMD (g/cm^2^)	1.123 ± 0.010	1.114 ± 0.10	1.115 ± 0.010	0.001 ± 0.002 [−0.003, 0.005]	0.654	0.700
Osteocalcin (μg/L)	20.3 ± 0.6	19.4 ± 0.7	19.1 ± 0.6	−0.26 ± 0.49 [−1.25, 0.72]	0.598	0.690
CTX (ng/L)	438.8 ± 17.7	439.7 ± 17.4	408.0 ± 15.8	−31.76 ± 11.15 [−53.92, −9.61]	0.005	**0.015**

CI = confidence interval; FDR = false discovery rate; BMD = bone mineral density; CTX = C‐terminal telopeptide type‐1 collagen.

Q* (FDR) means the adjusted level of significance after applying false discovery rate of 0.15.

We sought to see whether the magnitude of change in bone metabolism with resveratrol was influenced by baseline bone biomarkers. Indeed, there was a negative correlation between the treatment difference in CTX and baseline CTX levels (*r* = −0.208, *p* = 0.047). A stronger correlation was observed between the treatment difference in osteocalcin and baseline osteocalcin levels (*r* = −0.347, *p* < 0.001).

**Fig 3:**
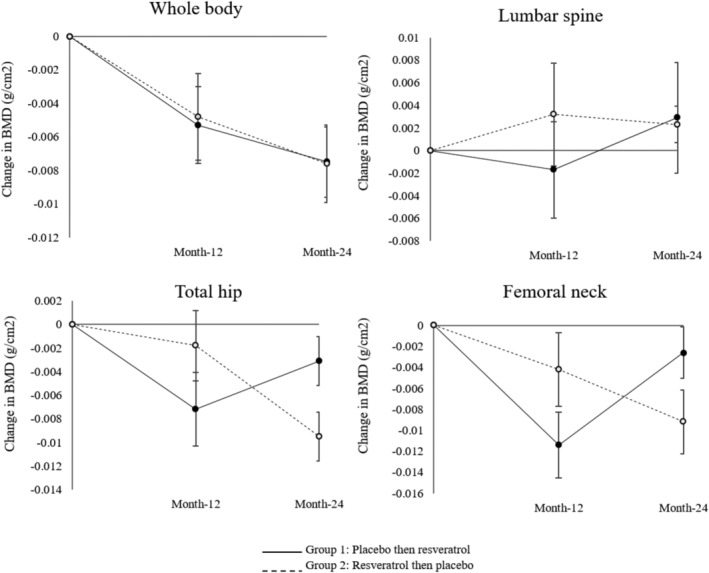
Change of bone mineral density (BMD) from baseline to month‐12 and month‐24 in the whole body, lumbar spine, total hip, and femoral neck for group 1 (placebo then resveratrol) and group 2 (resveratrol then placebo).

Fig. 3 shows the difference in BMD values of the lumbar spine, femoral neck, total hip, and whole body measured between baseline and month‐12 values, and month‐12 and month‐24 of the two groups at the end of each treatment phase. Group 1 was randomized to placebo treatment and group 2 received resveratrol in the first 12‐month arm; they then crossovered to the alternate treatment for another 12 months. In group 1, BMD of the lumbar spine, femoral neck, and total hip increased at month‐24 from their placebo values (at month‐12) after supplementing with resveratrol. In fact, the increase in BMD of the lumbar spine was higher than the baseline value. The resveratrol‐related BMD increase at the femoral neck and total hip did not return to their baseline values. In general, BMD values at the four sites decreased at month‐24 after placebo treatment in group 2; however, the decrease in BMD of the lumbar spine appeared to be attenuated after the crossover to placebo and remained higher than the baseline value.

### Treatment responsiveness in those supplemented with vitamin D or calcium or both

In the parallel analysis, the treatment difference from baseline in the spine BMD in the resveratrol group was greater in those who took calcium (+0.068 g/cm^2^) or both vitamin D and calcium (+0.036 g/cm^2^) compared with those who took neither (+0.002 g/cm^2^). The increase in spine BMD in those who took calcium only was greater than in those who took vitamin D only. The treatment difference from baseline in total hip BMD was also greater in the calcium only (+0.039 g/cm^2^) and vitamin D only (−0.020 g/cm^2^) groups compared with those who took neither (−0.006 g/cm^2^) (Supplemental Table S2). The factorial analysis confirmed the additive benefits for lumbar spine BMD of taking resveratrol together with vitamin D or calcium (Supplemental Table S3).

In the crossover comparison, the benefit of resveratrol over placebo on lumbar spine BMD was enhanced in those who regularly supplemented with both vitamin D and calcium (+0.028 g/cm^2^) compared with the calcium only (−0.026 g/cm^2^) group (Supplemental Table S4). Similarly, the women taking both vitamin D and calcium had an absolute increase of 0.027 g/cm^2^ in total hip BMD compared with the none group (+0.004 g/cm^2^), which resulted in a 1.3% reduction in hip fracture risk. Our factorial analysis did not show further additive effects (Supplemental Table S5).

### Correlations between treatment change in CVR and change in bone health parameters

The treatment increases in femoral neck *T*‐score correlated with increases in CVR (*R* = 0.323, *p* = 0.013). This correlation predominated in the group taking a vitamin D supplement only (*R* = 0.875, *p* = 0.004) but was still evident in the group taking neither vitamin D nor calcium (*R* = 0.297, *p* = 0.050). Treatment improvements in CVR did not correlate with all other bone parameters.

## Discussion

RESHAW is the longest clinical trial of resveratrol supplementation undertaken in postmenopausal women. After 12 months of supplementation with a low dose of resveratrol (75 mg twice daily), we found positive effects compared with placebo on bone density in the lumbar spine and neck of femur, which were accompanied by a reduction of plasma CTX. The increased BMD in the femoral neck resulted in improvement of *T*‐score and reduction in the 10‐year probability of major and hip fractures. Importantly, the improvement in femoral neck *T*‐score with resveratrol correlated with improvement in CVR to a hypercapnic challenge, which is a surrogate measure of cerebral vasodilator capacity and a potential general indicator of the ability of the microvasculature to deliver blood to tissues.

Three randomized controlled trials have been published reporting effects of resveratrol supplementation on bone health, but supplementation did not exceed 6 months in any of the studies. In a recent study, older adults with type 2 diabetes were supplemented for 6 months with resveratrol at a dose of 500 mg/d, which increased whole body BMD by 0.9% compared with placebo. However, no increase was observed at the lumbar spine or hip,^(^ ^14^ ^)^ which are critical sites advocated by the International Society for Clinical Densitometry (ISCD) for diagnosing osteoporosis based on the lowest DXA scan *T*‐score of either the femoral neck or lumbar spine.^(^ ^25^ ^)^ Similarly, in middle‐aged men with metabolic syndrome, increases in lumbar spine or total hip BMD after 16 weeks of resveratrol (500 mg twice daily) were no different to either placebo or a lower resveratrol dose (75 mg twice daily). Nonetheless, a 2.6% relative increase in the trabecular volume density of the lumbar spine was evident at the higher resveratrol dose, which correlated with a 16% increase in bone alkaline phosphatase, a marker of bone formation. Treatment changes in osteocalcin or CTX with resveratrol did not differ from those of the placebo.^(^ ^15^ ^)^ One study reported an increase in bone alkaline phosphatase but not markers of bone resorption in obese males supplemented for 4 weeks with 500 mg resveratrol daily.^(^ ^16^ ^)^

The results of our study in postmenopausal women are better than previously published studies of the bone‐protective effects of resveratrol undertaken in other target populations. Even in the parallel analysis, changes from baseline in BMD of the lumbar spine and femoral neck, which are the critical regions outlined by ISCD, as well as the total hip were significantly greater in the resveratrol arm than the placebo arm. In particular, the attenuation in the decline in femoral neck *T*‐score translates to reduction in hip fracture risk, which may be of clinical significance for those postmenopausal women at a lower risk of an osteoporotic fracture, enabling them to optimize their bone health with a dietary supplement. The within‐individual crossover comparisons confirmed these increases in BMD, apart from the total hip. Although we did not assess bone alkaline phosphatase or see an increase in osteocalcin levels, there was a relative reduction of 7.2% in the bone resorption marker, CTX, which none of the abovementioned studies had reported. In fact, participants with higher baseline CTX levels showed greater reductions of CTX levels with resveratrol. Likewise, we saw a stronger correlation between women with lower osteocalcin levels at baseline and the increase in osteocalcin with resveratrol relative to placebo, suggesting a greater potential for resveratrol to improve bone formation in those individuals at risk of rapid bone loss. It is worth noting that the effective dose of 75 mg for the bone health benefits observed in this study is 80 times higher than the average daily dietary intake. Findings from a Spain population where their main source of resveratrol intake is from drinking wine showed that the average dietary intake for trans‐resveratrol was 0.933 mg/d;^(26^ ^)^ hence, supplementation is necessary.

#### 4.1.1. Resveratrol versus genistein

Another phytoestrogen, the soy isoflavone genistein, has also been shown to benefit bone health in postmenopausal women. A clinical trial in 389 osteopenic postmenopausal women evaluated the effects of 56 mg/d of genistein (plus 400 IU of vitamin D) and found a remarkable BMD increase in the femoral neck (+0.023 g/cm^2^) and lumbar spine (+0.055 g/cm^2^) relative to placebo after 12 months of supplementation. BMD continued to increase in the second year of supplementation.^(^ ^10^ ^)^ Although the genistein‐induced BMD increase at the two skeletal sites appeared greater than in our study (femoral neck +0.009 g/cm^2^; lumbar spine +0.014 g/cm^2^), our participants were older (65 years old versus 54 years old) but had better bone health at baseline and reported fewer cases and severity of adverse events relating to resveratrol. As discussed above, individuals with poorer bone health are likely to show greater improvement with resveratrol; this may also be the case with genistein. Moreover, observations from a cross‐sectional study suggested benefits of soy isoflavones might be less pronounced in older compared with younger postmenopausal women.^(^ ^27^ ^)^ These factors might account for the lower absolute BMD increase observed with resveratrol compared with genistein.

### Resveratrol as a prophylactic in osteoporosis prevention

A longitudinal study of elderly residents living in the city of Dubbo in regional New South Wales of Australia reported an annual rate of decline of 0.96 ± 4.08% in BMD of the femoral neck and − 0.04 ± 3.09% in BMD of the lumbar spine in women >60 years old.^(^ ^28^ ^)^ Results from our parallel analysis showed a 1.0% decline in femoral neck BMD in the placebo group, in line with the population's annual rate of decline. In the resveratrol group, the rate of decline in femoral neck BMD from baseline was 0.34% after 12 months of supplementation, suggesting the potential to slow the decline through regular resveratrol supplementation.

In the parallel analysis, the change in lumbar spine BMD from baseline was −0.55% in the placebo group, which was higher than the published population average, but there was a + 0.46% increase in BMD from baseline in the resveratrol group. Owing to the positive association between high whole body BMD and incidence of radiographic osteoarthritis, independent of joint symptoms, the coexistence of spinal osteoarthritis and the large variation in spine BMD tends to obscure interpretations of treatment efficacy.^(^ ^29^ ^)^ We excluded data of participants with symptomatic spinal osteoarthritis or scoliosis from the analysis, thereby limiting this confounder. In osteoporotic postmenopausal women, anti‐fracture therapy with strontium ranelate (2 g/d) increased lumbar spine BMD by 13.6% over 2 years and reduced risk of new vertebral fracture by 49% in the first year of treatment relative to placebo.^(^ ^30^ ^)^ It is encouraging to see that the modest increase of 1.5% in spine BMD in our study is not limited to those with compromised bone health, as there was no difference in treatment responsiveness between participants with normal bone density and those with osteopenia/osteoporosis. The crossover study design allowed us to observe carryover effects after treatment cessation. Although we did not expect the resveratrol‐related BMD increase to persist after switching to placebo, the preservation of the BMD increase in the lumbar spine for 12 months after cessation of resveratrol is promising. Whether the increase in lumbar spine BMD with resveratrol is protective against future vertebral fracture in postmenopausal women with normal bone density is still unknown.

### Potential mechanisms of resveratrol for improving bone health

We believe that resveratrol can act through multiple mechanisms, particularly the estrogenic pathway, to exert positive effects on bone health in postmenopausal women. For instance, there was a slowing of BMD loss and deterioration of the trabecular structure of the femoral neck with resveratrol supplementation in ovariectomized rats.^(^ ^31^ ^)^ Resveratrol has an estrogen‐like effect on bone by increasing gene expression of osteoprotegerin, a protein that inhibits RANKL to counteract osteoclast differentiation at the precursor stage and activity at maturity.^(^ ^31^ ^)^ Reduction of bone loss in the vertebrae associated with increased bone formation markers, alkaline phosphatase and osteocalcin, was also reported in ovariectomized rat models treated with resveratrol.^(^ ^32^ ^)^ Resveratrol has also been shown in vitro to impair osteoclast formation by inhibiting RANKL‐induced nuclear factor‐kappa beta (NF‐κB) signaling cascade during osteoclastogenesis. Specifically, resveratrol has the capacity to suppress IκB‐alpha kinase phosphorylation and kinase activity and degradation to p65 dimers. In further inhibiting the RANKL‐induced NF‐κB osteoclastogenesis, NAD‐dependent deacetylase sirtuin‐1 (SIRT‐1) activity is upregulated by resveratrol, which in turn deactivates P300 acetyltransferase (an enzyme that promotes cell maturation and differentiation into specialized function) and acetylation of NF‐κB‐p65.^(^ ^33^ ^)^

Besides estrogen‐deficiency bone loss, senile osteoporosis also occurs concurrently in the later stage of postmenopausal osteoporosis in which pluripotent bone marrow mesenchymal stem cells preferentially differentiate into adipocytes over osteoblasts. Just 10 mg of resveratrol per kilogram of body mass per day for 10 weeks attenuated trabecular and cortical bone loss in the femoral neck of very old rats (22 months old).^(^ ^34^ ^)^ The preservation of the femur microarchitecture in a senile osteoporosis model is likely attributable to resveratrol‐induced enhancement of osteogenesis and inhibition of adipogenesis in human mesenchymal stem cells, primarily mediated through the SIRT‐1 pathway, with a small contribution from estrogenic signaling.^(^ ^35^ ^)^ However, resveratrol appeared to be detrimental to male rats in the early stage of aging after administration of 20 mg of resveratrol per kilogram of body mass per day for 12 weeks. The reduction in the trabecular bone volume of the tibia was accompanied by a decline in osteocalcin expression and an increase in resorption marker CTX, which was not offset by the slight increase in SIRT‐1 gene expression that mediates osteocalcin and alkaline phosphatase expressions.^(^ ^36^ ^)^

An important point to consider is the dose of resveratrol used to alleviate a particular health condition. Although Isabel and colleagues found that a 10 mg/kg dose of resveratrol attenuated senile osteoporosis,^(^ ^34^ ^)^ a subsequent study by Lee and colleagues found that the same dose exacerbated methotrexate‐induced reduction in growth plate thickness, trabecular bone volume, and metaphyseal primary spongiosa height, and increased marrow adiposity in young rats. In contrast, they found that a 1 mg/kg dose was sufficiently potent to negate the chemotherapy‐induced osteoclastic activity, thereby enabling normal endochondral bone growth and new bone deposition during treatment.^(^ ^37^ ^)^ Indeed, our dose–response studies have consistently shown that the maximal improvements in systemic and cerebral vasodilator function in humans with hypertension or type 2 diabetes were achieved with the lowest resveratrol dose used.^(^ ^19^, ^38^ ^)^ Taken together, the mechanisms underlying the important bone‐protective effect of resveratrol in elderly women may be via upregulating osteoprotegerin expression to reduce bone resorption activity, and simultaneously by inhibiting adipogenesis to favor osteoblast formation. Importantly, the optimal dose of resveratrol required to alleviate a particular health condition in a specific population should be determined in a dose–response study preceding an intervention trial.

Resveratrol‐induced improvement in vasodilator function is another plausible bone‐protective mechanism. Impaired blood flow in the lower extremities is associated with decreased BMD in the hip and ankle, independent of exogenous estrogen use.^(^ ^39^ ^)^ Administering eldecalcitol, a derivative of 25‐dihydroxy vitamin D_3_, in ovariectomized rats has resulted in an improvement in vascular function and increased BMD of lumbar vertebrae and femur.^(^ ^40^ ^)^ The evidence for resveratrol improving vascular function in humans is well established.^(^ ^19^, ^20^, ^41^ ^)^ In our recently published article reporting on the other outcomes of the RESHAW trial, CVR improved by 12% relative to placebo.^(^ ^22^ ^)^ To the best of our knowledge, this is the first clinical study to report a significant positive correlation between resveratrol‐induced improvements in microvascular function and femoral neck *T*‐score, supporting the importance of adequate perfusion for bone homeostasis.

### Additive benefit of resveratrol on bone health with vitamin D and/or calcium supplementation

Consumption of vitamin D and/or calcium supplements for maintaining bone health is widespread among the elderly population. Hence, we undertook a subgroup analysis to identify potential synergies with resveratrol in participants who took vitamin D and/or calcium supplements. The 12‐month parallel comparison showed that those who took calcium with or without vitamin D had a greater increase in lumbar spine BMD than those who took neither. Factorial analysis confirmed this observation. In the crossover comparison, those who took both calcium and vitamin D had a greater resveratrol‐induced increase in lumbar spine BMD than those taking calcium only. They also had a significantly greater increase in total hip BMD and reduction in hip fracture risk than those taking neither calcium nor vitamin D. We acknowledge that small sample sizes in the vitamin D and/or calcium groups limit interpretation of potential interactions. In addition, we did not collect data on the participants' serum 25‐hydroxyvitamin D levels or monitor dietary intake of calcium as the effect of resveratrol on bone health was a secondary outcome of this study. Hence, how these variables might have affected the responsiveness to resveratrol remains unknown. However, it is worth noting that the 12‐month duration of treatment phases in the crossover study design allowed us to assess within‐individual treatment effects on bone parameters without confounding effects of seasonal variation in 25‐dihydroxy vitamin D_3_ status, thus increasing the reliability of the observed resveratrol‐induced changes in parameters of bone health.

Notwithstanding the small sample sizes, there appears to be an additive benefit of resveratrol on BMD in women who took both vitamin D and calcium supplements. It is well established that vitamin D and calcium act synergistically to promote bone formation. Vitamin D assists bone formation by activating collagen type 1α, osteocalcin, and alkaline phosphatase gene transcription^(^ ^42^ ^)^ and, after conversion to 1,25‐hydroxyvitamin D, vitamin D facilitates calcium absorption in the gut and helps maintain adequate calcium concentration to promote bone mineralization. Meta‐analyses showed that supplementing with calcium plus vitamin D could reduce the risk of total and hip fractures by 15% and 30%, respectively,^(43^ ^)^ but vitamin D alone or calcium alone failed to produce meaningful effects on BMD.^(^ ^44^, ^45^ ^)^ The additive benefits of resveratrol with vitamin D and calcium may be attributable to the enhancement of vitamin D receptors in bone cells stimulated by resveratrol.^(^ ^46^ ^)^ As a phytoestrogen, resveratrol has a similar structure to estrogen, which has been shown to upregulate vitamin D receptors in osteoblast‐like cells to upregulate bone formation.^(^ ^47^ ^)^ The presence of vitamin D appears to amplify the biological effects of resveratrol. Compared with resveratrol alone, the combination of vitamin D plus resveratrol enhanced the intracellular concentration of resveratrol, increased activation of estrogen‐receptor‐β and vitamin D receptor expression, and boosted the bioavailability of resveratrol and vitamin D, thereby increasing their ability to rapidly cross the cell membrane and to reach target tissues.^(^ ^48^ ^)^ Furthermore, vitamin D also has a critical role in vascular function. Data from a larger community‐based study of older adults showed a significant association between vitamin D status and endothelial vasodilatation; however, this was only evident in older women.^(^ ^49^ ^)^ From our data, the vitamin D only group showed a strong correlation between the improvements in CVR and femoral neck *T*‐score with resveratrol, relative to other groups, suggesting that this unique combination may potentially improve vascular function and bone health synergistically. No clinical trials have been undertaken to investigate the synergistic benefit of resveratrol with vitamin D and/or with calcium on bone health. If our hypothesis is confirmed, it may offer a clinically meaningful and significant benefit to counteract postmenopausal osteoporosis.

In conclusion, low‐dose resveratrol supplementation significantly improved BMD of the lumbar spine and femoral neck and reduced the bone resorption marker, CTX, in postmenopausal women. The magnitude of benefit was greater for women with suboptimal bone metabolism. The resveratrol‐induced improvement in CVR and femoral neck *T*‐score suggests that improvement of the microcirculation may be an additional area to target in preventing postmenopausal osteoporosis. Furthermore, the benefits of resveratrol on spine and hip BMD appeared to be amplified in women who regularly consume vitamin D and calcium supplements. Additional clinical randomized controlled trials are warranted to test whether this unique combination can improve the vascular and osseous profiles of the elderly women.

## Disclosures

All authors state that they have no conflicts of interest.

## Author contributions

**Rachel Wong:** Conceptualization; data curation; formal analysis; funding acquisition; investigation; methodology; resources; supervision; validation; visualization; writing‐original draft; writing‐review and editing. **Jay Jay Thaung Zaw:** Data curation; investigation; validation; visualization; writing‐review and editing. **Cory Xian:** Methodology; supervision; validation; writing‐review and editing. **Peter Howe:** Conceptualization; funding acquisition; methodology; resources; supervision; validation; visualization; writing‐review and editing.

### Peer Review

The peer review history for this article is available at https://publons.com/publon/10.1002/jbmr.4115.

## Supporting information


**Supplemental Table S1.** Baseline Bone Health Status of Participants Who Did Not Take Versus Those Who Took Vitamin D and/or Calcium Supplements on a Regular Basis
**Supplemental Table S2.** Parallel Analysis of the Treatment Difference From Baseline of the Parameters of Bone Health in the Resveratrol Group Between Participants Who Were Not Supplemented and Those Who Took Vitamin D and/or Calcium Supplements
**Supplemental Table S3.** Factorial Analysis of the Treatment Difference From Baseline of the Parameters of Bone Health in the Resveratrol Group After 12 Months of Supplementation Between the Vitamin D and No Vitamin D Supplementation Group and Between the Calcium and No Calcium Supplementation Groups
**Supplemental Table S4.** Within‐Individual Comparisons in Treatment Change in the Parameters of Bone Health Between Those Who Were Not Supplemented and Those Who Took Vitamin D and/or Calcium Supplements Regularly
**Supplemental Table S5.** Factorial Analysis of the Within‐Individual Treatment Change in the Parameters of Bone Health Between Vitamin D and No Vitamin D Supplementation and Between Calcium and No Calcium Supplementation

## References

[1] Kalervo Väänänen H , Härkönen PL . Estrogen and bone metabolism. Maturitas. 1996;23:S65–S9. 8865143 10.1016/0378-5122(96)01015-8

[2] Kalaria R . Similarities between Alzheimer's disease and vascular dementia. J Neurol Sci. 2002;203–204:29–34. 10.1016/s0022-510x(02)00256-3 12417353

[3] Raisz LG . Pathogenesis of osteoporosis: concepts, conflicts, and prospects. J Clin Invest. 2005;115(12):3318–25. 16322775 10.1172/JCI27071 PMC1297264

[4] McGee‐Lawrence ME , Secreto FJ , Syed FA . Chapter 17—Animal models of bone disease‐B In Conn PM, ed. Animal models for the study of human disease. Boston, MA: Academic Press; 2013 pp 391–417.

[5] Riggs BL , Melton LJ . Evidence for two distinct syndromes of involutional osteoporosis. Am J Med. 1983;75(6):899–901. 6650542 10.1016/0002-9343(83)90860-4

[6] Wang L , Zhou F , Zhang P , et al. Human type H vessels are a sensitive biomarker of bone mass. Cell Death Dis. 2017;8(5):e2760. 28471445 10.1038/cddis.2017.36 PMC5520742

[7] Dandajena TC , Ihnat MA , Disch B , Thorpe J , Currier GF . Hypoxia triggers a HIF‐mediated differentiation of peripheral blood mononuclear cells into osteoclasts. Orthod Craniofac Res. 2012;15(1):1–9. 22264322 10.1111/j.1601-6343.2011.01530.x

[8] Utting JC , Robins SP , Brandao‐Burch A , Orriss IR , Behar J . Arnett TR Hypoxia inhibits the growth, differentiation and bone‐forming capacity of rat osteoblasts. Exp Cell Res. 2006;312(10):1693–702. 16529738 10.1016/j.yexcr.2006.02.007

[9] Laroche M , Pécourneau V , Blain H , et al. Osteoporosis and ischemic cardiovascular disease. Joint Bone Spine. 2017;84(4):427–32. 27838246 10.1016/j.jbspin.2016.09.022

[10] Marini H , Minutoli L , Polito F , et al. Effects of the phytoestrogen genistein on bone metabolism in osteopenic postmenopausal women: a randomized trial. Ann Intern Med. 2007;146(12):839–47. 17577003 10.7326/0003-4819-146-12-200706190-00005

[11] Marini H , Minutoli L , Polito F , et al. OPG and sRANKL serum concentrations in osteopenic, postmenopausal women after 2‐year genistein administration. J Bone Miner Res. 2008;23(5):715–20. 18433304 10.1359/jbmr.080201

[12] Beavers DP , Beavers KM , Miller M , Stamey J , Messina MJ . Exposure to isoflavone‐containing soy products and endothelial function: a Bayesian meta‐analysis of randomized controlled trials. Nutr Metab Cardiovasc Dis. 2012;22(3):182–91. 20709515 10.1016/j.numecd.2010.05.007

[13] Tou JC . Evaluating resveratrol as a therapeutic bone agent: preclinical evidence from rat models of osteoporosis. Ann N Y Acad Sci. 2015;1348(1):75–85. 26200189 10.1111/nyas.12840

[14] Bo S , Gambino R , Ponzo V , et al. Effects of resveratrol on bone health in type 2 diabetic patients. A double‐blind randomized‐controlled trial. Nutr Diabetes. 2018;8(1):51. 30237505 10.1038/s41387-018-0059-4 PMC6147949

[15] Ornstrup MJ , Harslof T , Kjaer TN , Langdahl BL , Pedersen SB . Resveratrol increases bone mineral density and bone alkaline phosphatase in obese men: a randomized placebo‐controlled trial. J Clin Endocrinol Metab. 2014;99(12):4720–9. 25322274 10.1210/jc.2014-2799

[16] Poulsen MM , Ornstrup MJ , Harslof T , et al. Short‐term resveratrol supplementation stimulates serum levels of bone‐specific alkaline phosphatase in obese non‐diabetic men. J Funct Foods. 2014;6:305–10.

[17] Guo X‐F , Li J‐M , Tang J , Li D . Effects of resveratrol supplementation on risk factors of non‐communicable diseases: a meta‐analysis of randomized controlled trials. Crit Rev Food Sci Nutr. 2018;58(17):3016–29. 28933578 10.1080/10408398.2017.1349076

[18] Wong RH , Raederstorff D , Howe PR . Acute resveratrol consumption improves neurovascular coupling capacity in adults with type 2 diabetes mellitus. Nutrients. 2016;8(7):425. 10.3390/nu8070425 PMC4963901 27420093

[19] Wong RHX , Nealon RS , Scholey A , Howe PRC . Low dose resveratrol improves cerebrovascular function in type 2 diabetes mellitus. Nutr Metab Cardiovasc Dis. 2016;26(5):393–9. 27105868 10.1016/j.numecd.2016.03.003

[20] Evans HM , Howe PRC , Wong RHX . Effects of resveratrol on cognitive performance, mood and cerebrovascular function in post‐menopausal women; a 14‐week randomised placebo‐controlled intervention trial. Nutrients. 2017;9(1):27. 10.3390/nu9010027 PMC5295071 28054939

[21] Klinge CM , Blankenship KA , Risinger KE , et al. Resveratrol and estradiol rapidly activate MAPK signaling through estrogen receptors α and β in endothelial cells. J Biol Chem. 2005;280(9):7460–8. 15615701 10.1074/jbc.M411565200

[22] Zaw JJT , Howe PRC , Wong RHX . Sustained cerebrovascular and cognitive benefits of resveratrol in postmenopausal women. Nutrients. 2020;12(3):828. 10.3390/nu12030828 PMC7146200 32244933

[23] Altman DG , Bland JM . Treatment allocation by minimisation. Br Med J. 2005;330(7495):843. 15817555 10.1136/bmj.330.7495.843 PMC556084

[24] Benjamini Y , Hochberg Y . Controlling the false discovery rate: a practical and powerful approach to multiple testing. J R Stat Soc B Methodol. 1995;57(1):289–300.

[25] International Society for Clinical Densitometry. 2019 ISCD Official Positions—Adult. Middletown, CT: ISCD; 2019.

[26] Zamora‐Ros R , Andres‐Lacueva C , Lamuela‐Raventos RM , et al. Concentrations of resveratrol and derivatives in foods and estimation of dietary intake in a Spanish population: European Prospective Investigation into Cancer and Nutrition (EPIC)‐Spain cohort. Br J Nutr. 2008;100(1):188–96. 18096094 10.1017/S0007114507882997

[27] Ho SC , Woo J , Lam S , Chen Y , Sham A , Lau J . Soy protein consumption and bone mass in early postmenopausal Chinese women. Osteoporosis Int. 2003;14(10):835–42. 10.1007/s00198-003-1453-9 12920508

[28] Jones G , Nguyen T , Sambrook P , Kelly PJ , Eisman JA . Progressive loss of bone in the femoral neck in elderly people: longitudinal findings from the Dubbo osteoporosis epidemiology study. BMJ. 1994;309(6956):691–5. 7950520 PMC2540818

[29] Hardcastle SA , Dieppe P , Gregson CL , Davey Smith G , Tobias JH . Osteoarthritis and bone mineral density: are strong bones bad for joints? Bonekey Rep. 2015;4:624. 25628884 10.1038/bonekey.2014.119 PMC4303262

[30] Meunier PJ , Roux C , Seeman E , et al. The effects of strontium ranelate on the risk of vertebral fracture in women with postmenopausal osteoporosis. N Engl J Med. 2004;350(5):459–68. 14749454 10.1056/NEJMoa022436

[31] Zhao H , Li X , Li N , et al. Long‐term resveratrol treatment prevents ovariectomy‐induced osteopenia in rats without hyperplastic effects on the uterus. Br J Nutr. 2014;111(5):836–46. 24073920 10.1017/S0007114513003115

[32] Lin Q , Huang Y‐M , Xiao B‐X , Ren G‐F . Effects of resveratrol on bone mineral density in ovarectomized rats. Int J Biomed Sci. 2005;1(1):76–81. 23674958 PMC3614578

[33] Shakibaei M , Buhrmann C , Mobasheri A . Resveratrol‐mediated SIRT‐1 interactions with p300 modulate receptor activator of NF‐kappaB ligand (RANKL) activation of NF‐kappaB signaling and inhibit osteoclastogenesis in bone‐derived cells. J Biol Chem. 2011;286(13):11492–505. 21239502 10.1074/jbc.M110.198713 PMC3064204

[34] Tresguerres IF , Tamimi F , Eimar H , et al. Resveratrol as anti‐aging therapy for age‐related bone loss. Rejuv Res. 2014;17(5):439–45. 10.1089/rej.2014.1551 24956408

[35] Tseng P‐C , Hou S‐M , Chen R‐J , et al. Resveratrol promotes osteogenesis of human mesenchymal stem cells by upregulating RUNX2 gene expression via the SIRT1/FOXO3A axis. J Bone Miner Res. 2011;26(10):2552–63. 21713995 10.1002/jbmr.460

[36] Lee AM , Shandala T , Nguyen L , et al. Effects of resveratrol supplementation on bone growth in young rats and microarchitecture and remodeling in ageing rats. Nutrients. 2014;6(12):5871–87. 25521206 10.3390/nu6125871 PMC4277004

[37] Lee AM , Shandala T , Soo PP , et al. Effects of resveratrol supplementation on methotrexate chemotherapy‐induced bone loss. Nutrients. 2017;9(3):255.

[38] Wong RHX , Howe PRC , Buckley JD , Coates AM , Kunz I , Berry NM . Acute resveratrol supplementation improves flow‐mediated dilatation in overweight/obese individuals with mildly elevated blood pressure. Nutr Metab Cardiovasc Dis. 2011;21(11):851–6. 20674311 10.1016/j.numecd.2010.03.003

[39] Vogt MT , Cauley JA , Kuller LH , Nevitt MC . Bone mineral density and blood flow to the lower extremities: the study of osteoporotic fractures. J Bone Miner Res. 1997;12(2):283–9. 9041062 10.1359/jbmr.1997.12.2.283

[40] Serizawa K , Yogo K , Tashiro Y , et al. Eldecalcitol prevents endothelial dysfunction in postmenopausal osteoporosis model rats. J Endocrinol. 2016;228(2):75. 26537128 10.1530/JOE-15-0332

[41] Wong RHX , Berry NM , Coates AM , et al. Chronic resveratrol consumption improves brachial flow‐mediated dilatation in healthy obese adults. J Hypertens. 2013;31(9):1819–27. 23743811 10.1097/HJH.0b013e328362b9d6

[42] van Leeuwen JP , van Driel M , van den Bemd GJ , Pols HA . Vitamin D control of osteoblast function and bone extracellular matrix mineralization. Crit Rev Eukaryot Gene Expr. 2001;11(1–3):199–226. 11693961

[43] Weaver CM , Alexander DD , Boushey CJ , et al. Calcium plus vitamin D supplementation and risk of fractures: an updated meta‐analysis from the National Osteoporosis Foundation. Osteoporosis Int. 2016;27(1):367–76. 10.1007/s00198-015-3386-5 PMC4715837 26510847

[44] Kahwati LC , Weber RP , Pan H , et al. Vitamin D, calcium, or combined supplementation for the primary prevention of fractures in community‐dwelling adults: evidence report and systematic review for the US preventive services task force. JAMA. 2018;319(15):1600–12.2967730810.1001/jama.2017.21640

[45] Bolland MJ , Grey A , Avenell A . Effects of vitamin D supplementation on musculoskeletal health: a systematic review, meta‐analysis, and trial sequential analysis. Lancet Diabetes Endocrinol. 2018;6(11):847–58. 30293909 10.1016/S2213-8587(18)30265-1

[46] Guo C , Sinnott B , Niu B , Lowry MB , Fantacone ML , Gombart AF . Synergistic induction of human cathelicidin antimicrobial peptide gene expression by vitamin D and stilbenoids. Mol Nutr Food Res. 2014;58(3):528–36. 24039193 10.1002/mnfr.201300266 PMC3947465

[47] Liel Y , Kraus S , Levy J , Shany S . Evidence that estrogens modulate activity and increase the number of 1,25‐dihydroxyvitamin D receptors in osteoblast‐like cells (ROS 17/2.8). Endocrinology. 1992;130(5):2597–601. 1315250 10.1210/endo.130.5.1315250

[48] Uberti F , Morsanuto V , Aprile S , et al. Biological effects of combined resveratrol and vitamin D3 on ovarian tissue. J Ovar Res. 2017;10:61. 10.1186/s13048-017-0357-9 PMC5602920 28915830

[49] Maggio M , De Vita F , Lauretani F , et al. Vitamin D and endothelial vasodilation in older individuals: data from the PIVUS study. J Clin Endocrinol Metab. 2014;99(9):3382–9. 24892991 10.1210/jc.2014-1536 PMC4154089

