# Supplementary material for: Regular Supplementation With Resveratrol Improves Bone Mineral Density in Postmenopausal Women: A Randomized, Placebo‐Controlled Trial
Source: J Bone Miner Res. 2020 Jul 14;35(11):2121–31. doi: 10.1002/jbmr.4115 (PMC7689937; doi:10.1002/jbmr.4115)
Supplement: Supplementary file 1 — Supplemental Table S1. Baseline Bone Health Status of Participants Who Did Not Take Versus Those Who Took Vitamin D and/or Calcium Supplements on a Regular Basis Supplemental Table S2. Parallel Analysis of the Treatment Difference From Baseline of the Parameters of Bone Health in the Resveratrol Group Between Participants Who Were Not Supplemented and Those Who Took Vitamin D and/or Calcium Supplements Supplemental Table S3. Factorial Analysis of the Treatment Difference From Baseline of the Parameters of Bone Health in the Resveratrol Group After 12 Months of Supplementation Between the Vitamin D and No Vitamin D Supplementation Group and Between the Calcium and No Calcium Supplementation Groups Supplemental Table S4. Within‐Individual Comparisons in Treatment Change in the Parameters of Bone Health Between Those Who Were Not Supplemented and Those Who Took Vitamin D and/or Calcium Supplements Regularly Supplemental Table S5. Factorial Analysis of the Within‐Individual Treatment Change in the Parameters of Bone Health Between Vitamin D and No Vitamin D Supplementation and Between Calcium and No Calcium Supplementation [file JBMR-35-2121-s001.docx]

|  | None (n=97) | Vitamin D only (n=16) | Calcium only  (n=2) | Both  (n=13) |
| --- | --- | --- | --- | --- |
| Age (years) | 63.9±0.7 | 69.5±1.8 | 72.5±5.0 | 67.9±2.0 |
| Lumbar spine |  |  |  |  |
| BMD (g/cm^2^) | 1.090±0.16 | 1.114±0.039* | 1.204±0.110 | 1.066±0.043 |
| T-score | -0.74±0.12 | -0.54±0.32 | 0.10±0.91 | -1.0±0.36 |
| Z-score | 0.58±0.13 | 1.08±0.33 | 1.80±0.92 | 0.38±0.36 |
| Neck of femur |  |  |  |  |
| BMD (g/cm^2^) | 0.902±0.012 | 0.857±0.028 | 0.956±0.079 | 0.825±0.031† |
| T-score | -0.98±0.08 | -1.30±0.20 | -0.60±0.58 | -1.52±0.23† |
| Z-score | 0.62±0.09 | 0.53±0.22 | 1.45±0.63 | 0.17±0.25 |
| Total hip |  |  |  |  |
| BMD (g/cm^2^) | 0.940±0.013 | 0.888±0.031 | 0.910±0.087 | 0.855±0.034 |
| T-score | -0.53±0.10 | -0.98±0.25 | -0.80±0.70 | -1.21±0.27 |
| Z-score | 0.57±0.11 | 0.39±0.26 | 0.70±0.74 | 0.03±0.29 |
| 10-year major fracture risk (%) | 4.7±0.4 | 6.2±1.1 | 5.5±2.9 | 6.8±1.2 |
| 10-year hip fracture risk (%) | 1.2±0.3 | 1.5±0.6 | 0.9±1.7 | 1.7±0.7 |
| Total body BMD (g/cm^2^) | 1.137±0.011 | 1.124±0.027 | 1.053±0.077 | 1.092±0.030 |
| Osteocalcin (ug/L) | 20.1±0.8 | 22.7±1.9 | No data | 20.4±2.1 |
| CTX (ng/L) | 455.1±19.8 | 463.0±47.1 | No data | 378.0±51.6 |
| BMD: bone mineral density. CTX: C-terminal telopeptide type-1 collagen. None: not taking vitamin D or calcium supplements. Both: vitamin D + calcium. *P<0.05 between none and vitamin D. †P<0.05 between none and both | | | | |

Supplementary Table 1 - Baseline bone health status of participants who did not take versus those who took vitamin D and/or calcium supplements on a regular basis.

Supplementary Table 2 - Parallel analysis of the treatment difference from baseline of the parameters of bone health of in the resveratrol group between participants who were not supplemented and those who took vitamin D and/or calcium supplements.

|  | None  (n=48) | Vitamin D only (n=11) | Calcium only  (n=1) | Both  (n=5) |
| --- | --- | --- | --- | --- |
| Lumbar spine |  |  |  |  |
| BMD (g/cm^2^) | 0.002±0.004 | 0.008±0.009 | 0.068±0.028^a,c^ | 0.036±0.014^b^ |
| T-score | 0.04±0.04 | 0.07±0.09 | 0.60±0.28^a^ | 0.30±0.14 |
| Z-score | 0.06±0.04 | 0.11±0.08 | 0.60±0.25^a^ | 0.35±0.12^b^ |
| Neck of femur |  |  |  |  |
| BMD (g/cm^2^) | -0.004±0.004 | 0.007±0.009 | -0.024±0.031 | -0.017±0.014 |
| T-score | -0.02±0.03 | 0.05±0.07 | -0.15±0.21 | -0.14±0.10 |
| Z-score | 0.02±0.04 | 0.07±0.08 | -0.15±0.24 | -0.14±0.12 |
| Total hip |  |  |  |  |
| BMD (g/cm^2^) | -0.006±0.003 | 0.011±0.007^d^ | 0.039±.022^a^ | -0.020±0.011^e^ |
| T-score | -0.05±0.03 | 0.12±0.06^d^ | 0.30±0.18 | -0.15±0.09^e^ |
| Z-score | 0.01±0.03 | 0.12±0.06 | 0.40±0.20 | -0.14±0.10^e,f^ |
| 10-year major fracture risk (%) | 0.07±0.20 | 0.24±0.45 | -2.20±1.41 | 0.65±0.71 |
| 10-year hip fracture risk (%) | -0.09±0.14 | 0.14±0.31 | -0.20±0.97 | 0.28±0.48 |
| Total body BMD (g/cm^2^) | -0.007±0.003 | -0.001±0.006 | 0.009±0.020 | 0.007±0.010 |
| Osteocalcin (ug/L) | -0.5±0.8 | -2.1±1.8 | -7.0±5.7 | -3.5±2.5 |
| CTX (ng/L) | -27.7±14.2 | -53.1±31.6 | No data | -9.7±51.7 |
| BMD: bone mineral density. CTX: C-terminal telopeptide type-1 collagen. None: not taking vitamin D or calcium supplements. Both: vitamin D + calcium. ^a^ P<0.05 between none and calcium only. ^b^ P<0.05 between none and both. ^c^ P<0.05 between vitamin D and calcium. ^d^ P<0.05 between none and vitamin D. ^e^ P<0.05 between calcium only and both. ^f^  P<0.05 between vitamin D only and both. | | | | |

Supplementary Table 3 – Factorial analysis of the treatment difference from baseline of the parameters of bone health of in the resveratrol group after 12 months of supplementation between the vitamin D and no vitamin D supplementation group, and between calcium and no calcium supplementation groups.

|  | No vitamin D (n=48) | Vitamin D  (n=16) | P-value | No calcium (n=59) | Calcium  (n=6) | P-value |
| --- | --- | --- | --- | --- | --- | --- |
| Lumbar spine |  |  |  |  |  |  |
| BMD (g/cm^2^) | 0.002±0.004 | 0.021±0.008 | 0.038* | 0.003±0.004 | 0.042±0.012 | 0.003* |
| T-score | 0.04±0.04 | 0.17±0.08 | 0.138 | 0.05±0.04 | 0.36±0.12 | 0.017* |
| Z-score | 0.06±0.04 | 0.21±0.07 | 0.060 | 0.07±0.03 | 0.40±0.11 | 0.006* |
| Neck of femur |  |  |  |  |  |  |
| BMD (g/cm^2^) | -0.004±0.004 | -0.001±0.007 | 0.770 | -0.002±0.004 | -0.018±0.012 | 0.204 |
| T-score | -0.02±0.03 | -0.01±0.05 | 0.858 | -0.01±0.03 | -0.14±0.09 | 0.182 |
| Z-score | 0.02±0.04 | 0.00±0.06 | 0.796 | 0.03±0.03 | -0.14±0.11 | 0.138 |
| Total hip |  |  |  |  |  |  |
| BMD (g/cm^2^) | -0.006±0.004 | 0.004±.006 | 0.137 | -0.003±0.003 | -0.008±0.010 | 0.628 |
| T-score | -0.05±0.03 | 0.06±0.05 | 0.058 | -0.02±0.03 | -0.06±0.09 | 0.656 |
| Z-score | 0.01±0.03 | 0.07±0.05 | 0.329 | 0.03±0.03 | -0.03±0.09 | 0.577 |
| 10-year major fracture risk (%) | 0.1±0.2 | 0.2±0.4 | 0.773 | 0.1±0.2 | 0.1±0.6 | 0.982 |
| 10-year hip fracture risk (%) | -0.1±0.1 | 0.2±0.3 | 0.393 | -0.1±0.1 | 0.2±0.4 | 0.608 |
| Total body BMD (g/cm^2^) | -0.007±0.003 | 0.002±0.005 | 0.150 | -0.006±0.003 | 0.007±0.009 | 0.173 |
| Osteocalcin (ug/L) | -0.5±0.8 | -2.9±1.4 | 0.144 | -0.8±0.8 | -4.1±2.3 | 0.175 |
| CTX (ng/L) | -27.7±14.1 | -41.2±26.8 | 0.657 | -32.0±12.8 | -9.7±51.4 | 0.676 |
| BMD: bone mineral density. CTX: C-terminal telopeptide type-1 collagen. None: not taking vitamin D or calcium supplements. Both: vitamin D + calcium. ^*^ P<0.05 | | | | | | |

Supplementary Table 4 – Within-individual comparisons in treatment change in the parameters of bone health between those who were not supplemented and those who took vitamin D and/or calcium supplements regularly.

|  | None  (n=88) | Vitamin D only (n=16) | Calcium only  (n=2) | Both  (n=11) |
| --- | --- | --- | --- | --- |
| Lumbar spine |  |  |  |  |
| BMD (g/cm^2^) | 0.017±0.004 | 0.009±0.010 | -0.026±0.024 | 0.028±0.011^a^ |
| T-score | 0.16±0.03 | 0.09±0.08 | -0.20±0.22 | 0.25±0.10 |
| Z-score | 0.15±0.03 | 0.06±0.08 | -0.25±0.20 | 0.26±0.09 |
| Neck of femur |  |  |  |  |
| BMD (g/cm^2^) | 0.005±0.003 | 0.011±0.006 | -0.016±0.018 | 0.005±0.008 |
| T-score | 0.04±0.02 | 0.08±0.05 | -0.10±0.13 | 0.06±0.06 |
| Z-score | 0.05±0.02 | 0.07±0.05 | -0.15±0.15 | 0.07±0.06 |
| Total hip |  |  |  |  |
| BMD (g/cm^2^) | 0.004±0.003 | 0.010±0.007 | -0.019±0.019 | 0.027±0.008^b^ |
| T-score | 0.03±0.02 | 0.07±0.04 | -0.15±0.11 | -0.05±0.05 |
| Z-score | 0.03±0.02 | 0.08±0.04 | -0.15±0.10 | 0.04±0.04 |
| 10-year major fracture risk (%) | 0.0±0.2 | 0.0±0.5 | 0.4±0.13 | -0.7±0.5 |
| 10-year hip fracture risk (%) | 0.0±0.2 | -0.1±0.5 | 0.2±0.14 | -1.3±0.6^b^ |
| Total body BMD (g/cm^2^) | 0.021±0.021 | 0.093±0.050 | -0.02±0.141 | 0.013±0.060 |
| Osteocalcin (ug/L) | -0.7±0.5 | -0.10±1.3 | No data | -4.0±1.6 |
| CTX (ng/L) | -31.5±13.7 | -82.0±33.2 | No data | -26.6±41.6 |
| BMD: bone mineral density. CTX: C-terminal telopeptide type-1 collagen. None: not taking vitamin D or calcium supplements. Both: vitamin D + calcium. ^a^ P<0.05 between calcium only and both. **^b^** P<0.05 between none and both. | | | | |

Supplementary Table 5 – Factorial analysis of the within-individual treatment change in the parameters of bone health between vitamin D and no vitamin D supplementation, and between calcium and no calcium supplementation.

|  | No vitamin D (n=88) | Vitamin D  (n=27) | P-value | No calcium (n=104) | Calcium  (n=13) | P-value |
| --- | --- | --- | --- | --- | --- | --- |
| Lumbar spine |  |  |  |  |  |  |
| BMD (g/cm^2^) | 0.017±0.004 | 0.015±0.007 | 0.811 | 0.016±0.003 | 0.018±0.010 | 0.861 |
| T-score | 0.15±0.03 | 0.14±0.06 | 0.839 | 0.15±0.03 | 0.16±0.08 | 0.893 |
| Z-score | 0.15±0.03 | 0.13±0.06 | 0.764 | 0.14±0.03 | 0.16±0.08 | 0.804 |
| Neck of femur |  |  |  |  |  |  |
| BMD (g/cm^2^) | 0.005±0.003 | 0.005±0.005 | 0.961 | 0.006±0.003 | -0.002±0.007 | 0.246 |
| T-score | 0.03±0.02 | 0.05±0.03 | 0.685 | 0.04±0.02 | 0.00±0.05 | 0.433 |
| Z-score | 0.05±0.02 | 0.04±0.04 | 0.940 | 0.05±0.02 | 0.00±0.05 | 0.418 |
| Total hip |  |  |  |  |  |  |
| BMD (g/cm^2^) | 0.003±0.003 | 0.015±0.005 | 0.044 | 0.004±0.003 | 0.018±0.007 | 0.056 |
| T-score | 0.03±0.02 | 0.01±0.03 | 0.692 | 0.03±0.02 | -0.04±0.04 | 0.081 |
| Z-score | 0.03±0.02 | 0.05±0.03 | 0.577 | 0.04±0.01 | 0.03±0.04 | 0.745 |
| 10-year major fracture risk (%) | 0.0±0.2 | -0.3±0.4 | 0.532 | 0.0±0.2 | -0.4±0.5 | 0.479 |
| 10-year hip fracture risk (%) | 0.0±0.2 | -0.5±0.4 | 0.194 | 0.0±0.2 | -0.9±0.5 | 0.103 |
| Total body BMD (g/cm^2^) | 0.020±0.021 | 0.089±0.037 | 0.106 | 0.031±0.019 | 0.081±0.052 | 0.366 |
| Osteocalcin (ug/L) | -0.6±0.6 | -1.5±1.0 | 0.478 | -0.7±0.5 | -1.5±1.5 | 0.642 |
| CTX (ng/L) | -30.5±13.5 | -52.5±25.3 | 0.445 | -38.2±12.6 | -11.1±36.8 | 0.489 |
| BMD: bone mineral density. CTX: C-terminal telopeptide type-1 collagen. None: not taking vitamin D or calcium supplements. Both: vitamin D + calcium. | | | | | | |
